# Rhizobacteria‐Induced Systemic Priming Against Fungal Pathogens Involves Hydroxycinnamic Acid Amides

**DOI:** 10.1111/pce.70495

**Published:** 2026-03-30

**Authors:** Mackenzie Eli William Loranger, Wolfgang Moeder, Hyunsuh Lee, David K. Liscombe, Eric Déziel, Keiko Yoshioka

**Affiliations:** ^1^ Department of Cell and Systems Biology University of Toronto Toronto Ontario Canada; ^2^ Vineland Research & Innovation Centre Vineland Station Ontario Canada; ^3^ Department of Biological Sciences Brock University St. Catharines Ontario Canada; ^4^ Centre Armand‐Frappier Santé Biotechnologie Institut National de la Recherche Scientifique (INRS) Laval Quebec Canada

**Keywords:** Bacillus velezensis, hydroxycinnamic acid amides, induced systemic resistance, ISR, metabolomics, plant immunity, Pseudomonas defensor WCS374r, rhizosphere, Solanum lycopersicum

## Abstract

The rhizosphere, a narrow region of soil surrounding roots, contains diverse microorganisms with a composition that is distinct from the surrounding soil. Some rhizosphere bacteria can trigger a heightened state of immunity in the whole plant, termed Induced Systemic Resistance (ISR). To understand the mechanisms behind this enhanced resistance we investigated the metabolic profile of tomato plants treated with *Pseudomonas defensor* WCS374r and *Bacillus velezensis* VFb49, bacterial strains know to elicit ISR in tomato, using an untargeted approach, from which we identified a set of differentially accumulated metabolites (DAMs). We show that some of these metabolites—the hydroxycinnamic acid amides (HCAAs) caffeoyl putrescine and feruloyl putrescine—exhibit direct antimicrobial properties against two fungal pathogens, *Botrytis cinerea* and *Fusarium virguliforme*. Further, we show that in *Arabidopsis thaliana* agmatine coumaroyl transferase (*AtACT*) is induced by both bacteria and is required for resistance to *B. cinerea*. The apoplastic location of these compounds is also crucial for their function as the multidrug and toxin extrusion (MATE) transporter, *AtDTX18*, is essential for the resistance phenotype. Overall, this work offers novel insights into the mechanism of ISR, suggesting that the accumulation of HCAAs is one of the factors conferring enhanced resistance to pathogen infection.

## Summary statement

Untargeted metabolomics in tomato leaves revealed that immunity‐inducing root bacteria elevate the hydroxycinnamic acid amides caffeoyl‐ and feruloyl‐putrescine. These metabolites exhibit antifungal activity, revealing a previously unrecognised defence mechanism in ISR.

## 1 Introduction

The rhizosphere is the interface between the plant root and the surrounding soil. This narrow region around the roots is an area of intense interaction between the plant and the microorganisms in the soil (Trivedi et al. 2020). Plant root exudates shape the microbial composition of this area, which differs from the surrounding soil (Haichar et al. 2008). The composition of these exudates can change under abiotic and biotic stress, which can lead to the recruitment of select microbes (Rolfe et al. 2019; Goossens et al. 2023). This pool of microorganisms is referred to as the rhizosphere microbiota; the total pool of genetic material is known as the microbiome (Berendsen et al. 2012; Pieterse et al. 2014). Plants, in turn, often benefit from these rhizobacteria, leading to enhanced growth, nutrient and water uptake and increased tolerance to abiotic and biotic stresses. Thus, many rhizobacteria are known as Plant Growth Promoting Rhizobacteria, or PGPR (Prasad et al. 2019; Trivedi et al. 2020).

Beneficial rhizobacteria can protect plants from pathogenic microorganisms in at least two ways; first, by direct competition, either limiting resources or through anti‐microbial secretions between microorganisms. Second, certain rhizobacteria can trigger an enhanced state of immunity in the whole plant, which is known as Induced Systemic Resistance (ISR) (Pieterse et al. 2014). ISR has been demonstrated across a varied range of agriculturally relevant crop species, including tomato (Suresh et al. 2022), rice (De Vleesschauwer et al. 2008) and wheat (Dutilloy et al. 2024). Some bacterial strains are unique to specific plant species or even varieties, while others can confer resistance to a diverse set of plant hosts (Berendsen et al. 2015). Many PGPR and ISR‐inducing rhizobacteria belong to the genera *Bacillus* and *Pseudomonas* (Beneduzi et al. 2012). Among *Bacilli*, strains of *B. velezensis*, belonging to the operational group *B. amyloliquefaciens*, are widely used in agriculture (Rabbee et al. 2019; Ngalimat et al. 2021). The *Pseudomonas* genus includes two of the best‐characterised ISR inducing bacteria, *Pseudomonas simiae* WCS417, which was originally isolated from wheat roots (Pieterse et al. 2020), and *Pseudomonas defensor* WCS374r, which was isolated from potato roots (Berendsen et al. 2015).

Over the last two decades, significant progress has been made in understanding the molecular mechanisms of ISR. For example, it is now clear that some beneficial bacteria can suppress local host defence responses in the root to allow for colonisation (Stringlis et al. 2018; Teixeira et al. 2021). At the same time, they also release lipopeptides, siderophores, and volatile organic compounds (VOCs) that prime the plant for faster and stronger activation of defence responses, not only in the root but in the whole plant (Pieterse et al. 2014; Pescador et al. 2022; Salwan et al. 2023). In *Arabidopsis thaliana*, ISR activation requires the expression of the *AtMYB72* transcription factor in the root, which is considered an ISR marker gene (Pozo et al. 2008; Zamioudis et al. 2015). AtMYB72 triggers increased expression of the β‐glucosidase *AtBGLU42*, which facilitates the release of the coumarin scopoletin into the rhizosphere (Zamioudis et al. 2014; Stassen et al. 2021). This primed state allows for the plant to respond faster and stronger to pathogen attacks, with little to no growth penalties.

The mechanism conferring the primed state in the above ground tissues is still not well understood. Early microarray analyses in Arabidopsis suggested little to no changes in gene expression in foliar tissue of ISR‐induced plants under pre‐stress conditions, while a heightened transcriptional response was observed upon pathogen infection (Verhagen et al. 2004; Van Der Ent et al. 2009). Studies in Arabidopsis showed that ISR is mainly dependent on jasmonic acid (JA) and ethylene (ET) signalling, rather than salicylic acid (SA) (Pieterse et al. 1998; Van Der Ent et al. 2009). Indeed, motif enrichment analysis of primed genes, i.e., genes that responded more strongly in ISR‐primed plants, suggested that they are likely under the control of the transcription factor AtMYC2, a positive regulator of JA‐mediated defence genes (Pozo et al. 2008). Another transcription factor associated with ISR is the AP2/ERF transcription factor AtORA59, which is regulated by JA and ET (Pangesti et al. 2016).

AtORA59 regulates expression of the agmatine coumaroyl transferase gene (*AtACT*) (Li et al. 2018a) which encodes the enzyme that catalyzes the last reaction in the biosynthesis of hydroxycinnamic acid amides (HCAAs). Also known as phenolamides, HCAAs are found throughout the plant kingdom and play important roles in plant growth, development, and defence against biotic and abiotic stresses (Liu et al. 2022). HCAAs are synthesised through the condensation of hydroxycinnamic acid (HCA) derivatives with monoamines or polyamines (e.g., putrescine and spermidine) (Bassard et al. 2010; Liu et al. 2022). HCAs like coumaric acid, ferulic acid, and caffeic acid are synthesised from phenylalanine via the phenylpropanoid pathway (Bassard et al. 2010). They are then converted to HCA‐CoA thioesters, and these phenolic acid donors are transferred to the polyamine acceptors by hydroxycinnamoyl transferases. Common acceptors are the polyamines agmatine, putrescine, and spermidine, but also the monoamine tyramine, or quinic acid (Liu et al. 2022). HCAAs levels in plants increase upon biotic or abiotic stresses (Muroi et al. 2009; López‐Gresa et al. 2016; Mashabela et al. 2023b).

Untargeted metabolomics has emerged as a powerful tool in the study of plant immunity. However, ISR‐related metabolomics has been largely focused on post‐challenge responses (Carlson et al. 2019; Mashabela et al. 2023b; Hossain et al. 2024), rather than exploring the changes that occur in the primed plant prior to pathogen infection. Given this knowledge gap, we investigated the metabolic profile of ISR‐primed plants treated with two different bacteria: the Gram‐negative *P. defensor* WCS374r (Berendsen et al. 2015) and the Gram‐positive *Bacillus velezensis* VFb49 (Loranger et al. 2025) using an untargeted approach, from which we identified a subset of differentially accumulated metabolites (DAMs) compared to non‐primed plants. We demonstrate by both in vitro and in planta assays that some of these metabolites, particularly two from the HCAA class of compounds, exhibit direct antimicrobial properties. Overall, this work offers novel insights into the primed state of plants following ISR induction, suggesting that some of the enhanced resistance is directly attributed to the accumulation of this class of metabolites.

## 2 Results

### 2.1 Strain Specific Changes in the Tomato Metabolic Profile

To analyze metabolic changes upon priming with ISR‐inducing bacteria, tomato (*Solanum lycopersicum*) cv. ‘Glamour’ plants were treated with two ISR‐inducing strains, *B. velezensis* VFb49 (hereafter called VFb49; (Loranger et al. 2025)) and *P. defensor* WCS374r (hereafter called WCS374r; Berendsen et al. 2015). MgSO_4_ and *Escherichia coli*, were used as controls. The inclusion of *E. coli* was used to identify metabolites that accumulate in response to bacteria in general. Tomato seedlings were added to Jiffy pots that had been soaked in bacterial solutions; above ground tissue was then collected 2‐week posttreatment. We have previously shown that plants treated with VFb49 and WCS374r display enhanced resistance against *B. cinerea* at that time point (Loranger et al. 2025). Untargeted metabolomics of plant tissues was performed in positive ionisation mode (ESI+), resulting in the detection of 4065 mass spectrometric features.

**Figure 1:**
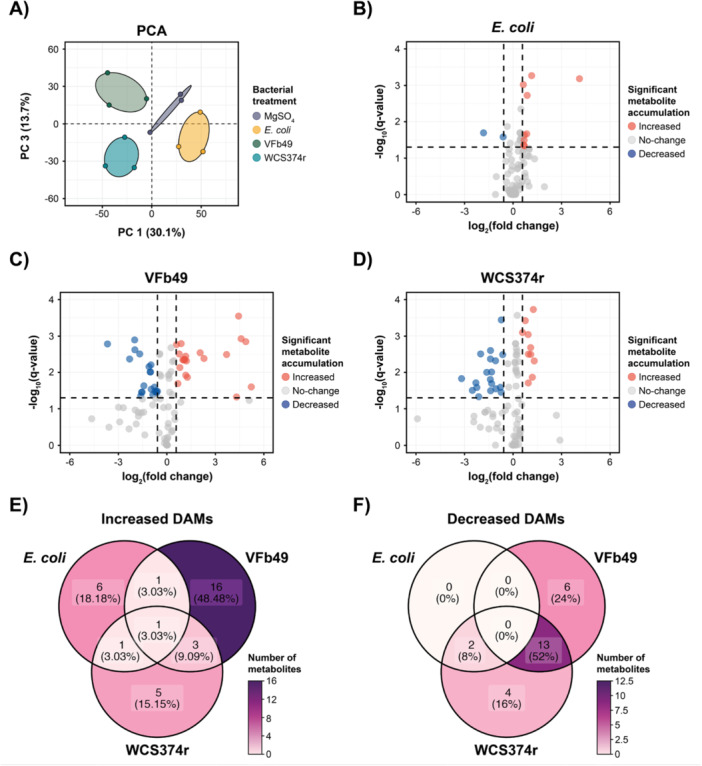
Comparison of metabolic profiles of tomato plants after bacterial treatments. (A) Principal component analysis (PCA) plot of the 1st and 3rd principal components. PCA was conducted on the complete set of detected metabolic features (4065 features), prior to statistical filtering. Ellipses drawn connecting the three replicates of each treatment. (B–D) Volcano plots showing the distribution of differentially accumulated metabolites after a two‐stage statistical analysis. The 4065 features were first screened using a one‐way ANOVA, from which 94 of them showed significant variation across the treatment groups. The *p*‐values were adjusted using the Benjamini & Hochberg (BH) FDR method, with *q*‐values < 0.05 being set as the cut‐off. These metabolites were then processed with Dunnett's post hoc pairwise comparison against the MgSO_4_ control. Fold change cutoff was set to 0.58 (log_2_FC(1.5)) and adjusted *p*‐value (*q*‐value) to 0.05 (‐log10(*q*‐value) = 1.301) (*n* = 3). (E and F) Venn diagrams of DAMs overlapping between *E. coli*, VFb49 and WCS374r after volcano plot filtering. Fill colour indicates the number of metabolites represented in the intersection.

To assess the plant metabolic changes in the context of ISR priming, we first investigated strain‐specific signatures using an unsupervised principal component analysis (PCA). Principal component 1 (PC1), which represents 30.1% of the total variance, shows a clear separation of the ISR inducing treatments (VFb49 and WCS374r) from the non‐ISR controls (MgSO_4_ and *E. coli*). Principal component 3 (PC3), which represents 13.7% of the total variance, distinguishes the two ISR strains from each other, suggesting that, while ISR inducers elicit overlapping metabolic changes (as seen in PC1), they also retain distinct profiles (Figure 1A). These differences likely reflect different colonisation patterns and bioactive metabolite production between *B. velezensis* and *P. defensor* (Xue et al. 2025). PC2 explained 19.4% of the total variation, however, it was related to differences among biological replicates of the same treatment.

DAMs for each treatment were determined based on their log₂ fold change (log₂FC) relative to the MgSO₄ control and their adjusted *p*‐value (*q*‐value) following a two‐stage approach, consisting of an initial one‐way ANOVA followed by a post hoc Dunnett's test. The fold change cutoff was set to a 1.5 change compared to the control (abs(log2FC) > 0.58) and an adjusted *p*‐value of 0.05. This filtering resulted in 40 DAMs in VFb49‐treated plants (21 increased, 19 decreased), 29 in WCS374r‐treated plants (10 increased, 19 decreased), and 11 DAMs in *E. coli*‐treated plants (9 increased, 2 decreased) (Figure 1B–D; Supporting Information S2: Tables S1–S3). A total of 16 DAMs (3 increased, 13 decreased) were shared between VFb49 and WCS374r. A single DAM was found to overlap between all 3 treatments (Figure 1E,F, Supporting Information S2: Table S4).

To determine the contribution of specific DAMs to the overall metabolic response, we first ranked all their associated DAMs according to their PC3 loading values, with higher absolute values indicating a greater contribution to strain‐specific metabolic separation (Supporting Information S2: Tables S1 and S2). In addition to a greater number of DAMs, VFb49‐treated plants exhibited a larger proportion of DAMs with absolute PC3 loading values greater than 0.5, suggesting that VFb49 treatment induced more extensive metabolic changes and resulted in stronger separation from the other treatments, when compared to WCS374r‐treated plants. VFb49 had a total of 22 DAMs that were either unique to the treatment or that showed an opposite dynamic to the other treatments (16 increased, 6 decreased), WCS374r had a total of 9 uniquely regulated DAMs (5 increased, 4 decreased), and *E. coli* resulted in 6 unique DAMs, all of which increased in abundance (Figure 1E,F).

Among the subset of VFb49‐specific DAMs that could be identified (Supporting Information S2: Table S1), indole‐3‐butyric acid, a precursor of indole‐3‐acetic acid (IAA) that also displays auxin activity in plants (Damodaran and Strader 2019), was increased. 7,8‐diaminopelargonate (DAPA), an intermediate in biotin biosynthesis was also increased (Knowles 1989). The list of WCS374r‐specific DAMs is shorter, and few exhibited strong PC3 loading values. Only four DAMs had an absolute value greater than 0.5 (Supporting Information S2: Table S2). Only one WCS374r specific compound could be putatively annotated: Dihydroactinidiolide, a volatile terpene that is also induced in tomatoes by *Fusarium oxysporum* (Hernández‐Aparicio et al. 2021).

### 2.2 Shared Metabolic Changes Associated With ISR Priming

**Figure 2:**
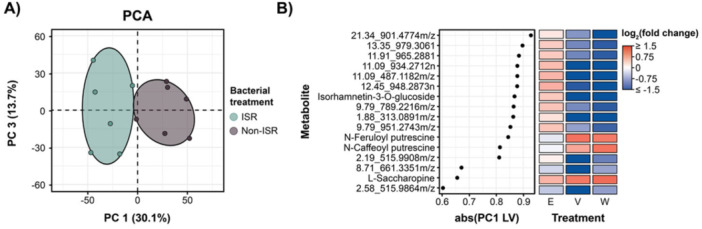
Shared ISR metabolic signature of VFb49 and WCS374r. (A) PCA plot with replicates coloured as either ISR inducers or Non‐ISR inducers (three replicates for each treatment). We visualised PC1 and PC3, which explained 30.1% and 13.7% of the total variance, respectively, while PC2 explained 19.4%, which was related to variation among biological replicates of the same treatment. (B) Heat map of all ISR overlapping metabolic features that changed in the same manner (16), values are log_2_FC compared to MgSO_4_ treatment. Metabolites were sorted based on their absolute loading scores from principal component 1 of the PCA. E—*E. coli*, V—VFb49, W—WCS374r. Sample size for each treatment: *n* = 3.

We defined the metabolic ISR signature as the 16 metabolites that overlapped between VFb49 and WCS374r. PC1 clearly separated ISR (VFb49 and WCS374r) and non‐ISR inducing treatments (MgSO_4_ and *E. coli*) (Figure 2A) and, therefore, the absolute loading values from this component was used to rank the ISR related DAMs. The loading values for these DAMs were generally high, with most exceeding 0.8, suggesting that these metabolites strongly contribute to defining the outcome of the treatments. These metabolites showed either no significant change or an inverse change in response to the *E. coli* treatment as visualised by the log2FC heat map (Figure 2B). Interestingly, two DAMs belonging to the HCAAs were increased, *N*‐caffeoyl putrescine (CP) and *N*‐feruloyl putrescine (FP). The third DAM that increased was l‐saccharopine, an intermediate of lysine metabolism, which has been connected to several forms of abiotic stresses (Wang et al. 2019; Lu et al. 2023; Li et al. 2024). l‐saccharopine can also be a precursor to pipecolic acid, a well‐studied defence signalling molecule involved in systemic priming (Arruda and Barreto 2020). Of the decreased DAMs, only the flavonoid isorhamnetin 3‐O‐glucoside could be annotated.

### 2.3 Changes in the Dynamics of HCAA Biosynthesis

**Figure 3:**
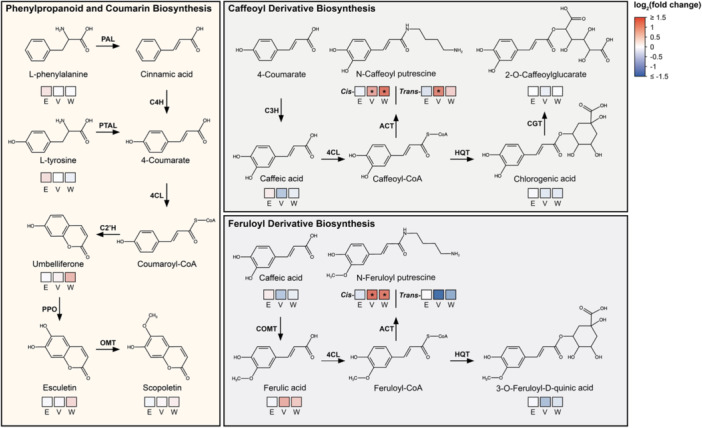
Changes in Caffeoyl‐ and Feruloyl‐putrescine metabolic pathways. Model of the primary phenylpropanoid biosynthesis pathway, highlighting branches leading to caffeoyl and feruloyl derivatives. Coloured squares denote log_2_FC in accumulation compared to the MgSO_4_ treatment. An asterisk (*) within the box denotes a statistically significant change that also meets the log_2_FC cutoff (0.58, log_2_FC(1.5)). Compounds without log_2_FC data were not detected. PAL: l‐phenylalanine ammonia‐lyase, C4H: Cinnamate 4‐hydroxylase, PTAL: Bifunctional l‐phenylalanine/l‐tyrosine ammonia‐lyase, 4CL: 4‐hydroxycinnamate:CoA ligase, C2'H: p‐Coumaroyl CoA 2'‐hydroxylase, PPO: Polyphenol oxidase, OMT: O‐methyltransferase, C3H: 4‐Coumarate 3‐hydroxylase, ACT: Agmatine coumaroyl transferase, HQT: Hydroxycinnamoyl CoA quinate transferase, CGT: Chlorogenate:glucarate O‐(hydroxycinnamoyl)transferase, COMT: caffeate/5‐hydroxyferulate 3‐O‐methyltransferase. [Color figure can be viewed at wileyonlinelibrary.com]

Among HCAAs, only CP and FP showed an increase in relative abundance (Figure 3), with CP being nearly 50‐fold more abundant than FP (Supporting Information S1: Figure S2). For CP, MS fragmentation data were obtained for the 2.36_251.1391 m/z feature. The initial peak of 251.1391 m/z corresponds to the monoisotopic [M + H] ion of CP (251.1388 m/z), while the 234.1129 m/z fragment matches to the loss of the terminal amine group, and the 163.0396 m/z fragment likely represents the caffeoyl fragment after loss of putrescine, strongly supporting the annotated identity (Supporting Information S1: Figure S1). Our MS chromatogram and normalised abundance analysis revealed both trans and cis isomers of CP and FP (Supporting Information S1: Figure S2). For CP, a twofold increase was observed for both isomers, although the increase in trans‐CP was not statistically significant in WCS374r‐treated plants. For FP, similarly, a statistically significant twofold increase in cis‐FP was detected in both treatments. Interestingly, a twofold, non‐significant decrease in the trans‐FP isomer was observed in both treatments (Supporting Information S1: Figure S2). However, this trend may be reflection of the very low levels of FP compared to CP (10–50 times lower) and is unlikely to have biological relevance. Fragmentation data for FP could not be obtained; it was, therefore, assigned a Level 2 confidence score according to the Metabolomics Standards Initiative (MSI) (Sumner et al. 2007). None of the other identified HCAA derivatives changed significantly, including the precursors caffeic acid and ferulic acid as well as caffeoyl quinic acid (chlorogenic acid), 2‐O‐caffeoyl glucarate and feruloyl quinic acid (Figure 3, Supporting Information S2: Table S5), suggesting that the accumulation of these specific compounds may be a key feature of the ISR metabolic signature.

### 2.4 The Role of HCAAs in ISR

**Figure 4:**
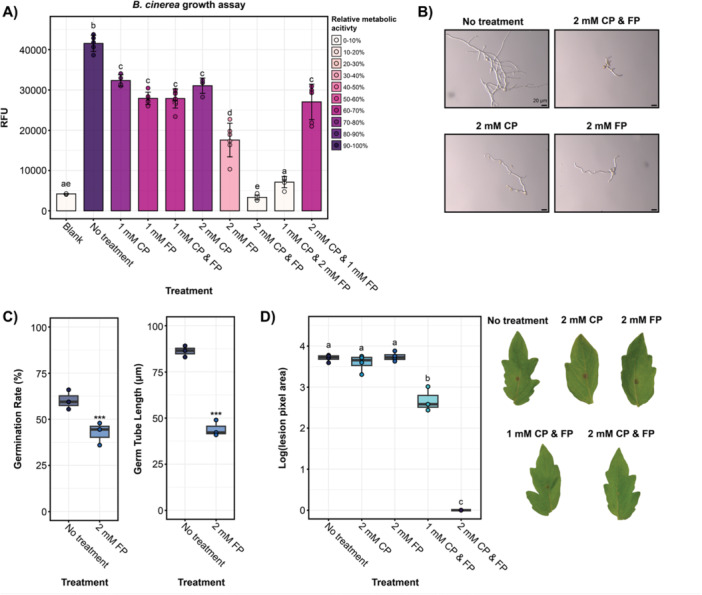
In vitro and in‐planta activity of HCAAs against B. cinerea. (A) Bar graph of resazurin‐based metabolic analysis of *B. cinerea* grown with various combinations and concentrations of FP and CP. Bar colour indicates % relative metabolic activity, with lighter colours indicating less activity, suggesting stronger impact of the treatment (each bar represents the mean ± SD, *n* = 5–6; one‐way ANOVA followed by a Tukey's HSD, different letters denote significant differences between treatment). (B) Images of individual *B. cinerea* spores 24 h after incubation with specified CP and FP treatments. (C) Boxplot quantification of the effect of 1 mM FP on *B. cinerea* spore germination rate and germ tube length (*n* = 3; ***p < 0.001, **p < 0.01, *p < 0.05. ANOVA followed by post hoc Dunnett's test). The boxes represent the 25th to 75th percentile, with the 50th percentile indicated by the black line (median). Whiskers extend to 1.5 times the interquartile range (IQR). Points are an overlay of individual data values for the replicates. (D) Lesion development in *Solanum lycopersicum* after infection with *B. cinerea*. Spores with various combinations of FP and CP were inoculated on leaves. Boxplot of lesion size (left), lesions on leaves after 4 days (right) (*n* = 3–4). One‐way ANOVA followed by a Tukey's HSD, different letters denote significant differences between treatment). [Color figure can be viewed at wileyonlinelibrary.com]

Since two of the three DAMs that increased following ISR priming were HCAAs, we investigated their role in ISR. Given that various HCAAs have direct anti‐microbial activity against a wide range of fungal and bacterial pathogens (Muroi et al. 2012; Dobritzsch et al. 2016; Liu et al. 2022), we investigated whether CP or FP had any anti‐fungal activity towards *B. cinerea*, the pathogen we originally used to identify the ISR activity of VFb49 (Loranger et al. 2025). Using a resazurin reduction assay to quantify metabolic activity, we observed that both FP and CP had in vitro inhibitory effects against *B. cinerea* at both 1 and 2 mM concentrations, with FP (64% at 2 mM) having a notably stronger effect than CP (28% at 2 mM) (Figure 4A). Unexpectedly, these two HCAAs also functioned in a synergistic manner, where 2 mM FP and CP used together resulted in almost complete inhibition of fungal metabolic activity. However, FP clearly had a dominant effect as 2 mM CP + 1 mM FP did not lead to increased inhibition compared to 1 mM FP alone. Further, microscopic analysis of spore germination in the presence of the compounds revealed that both compounds affected germ tube length (Figure 4B). We quantified the effect of FP on spore germination and germ tube length: 2 mM FP resulted in a nearly halving of germ tube length, reducing the average from 86 to 44 µm and a 17% reduction in germination rate (60.26% vs. 42.7%; Figure 4C). These results are comparable to the previously demonstrated anti‐fungal ability of another HCAA, p‐coumaroyl agmatine, against *B. cinerea* (Muroi et al. 2012).

To assess the in‐planta efficacy of these compounds, various combinations of CP and FP were added to *B. cinerea* spore suspensions, which were then inoculated onto tomato leaves. Lesions development was assessed 4‐day postinoculation. Both compounds showed minimal activity when applied individually at a concentration of 2 mM. However, when combined at either 1 mM or 2 mM, they significantly reduced lesion development, indicating strong synergistic activity (Figure 4D). Notably, the 2 mM combination treatment completely suppressed lesion formation.

**Figure 5:**
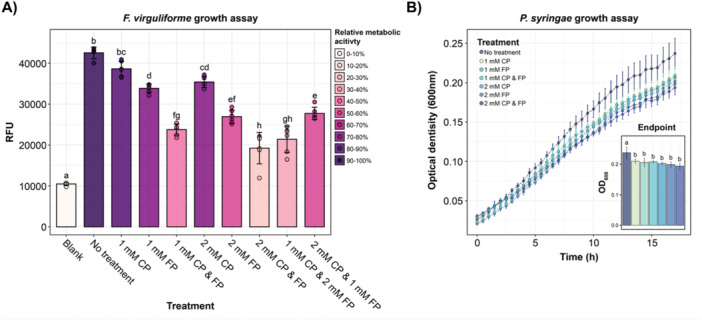
Anti‐microbial activity of HCAAs against *Fusarium virguliforme* and *Pseudomonas syringae* DC3000. (A) Bar graph of resazurin‐based metabolic analysis of *Fusarium virguliforme* grown with various combinations and concentration of the CP and FP. Bar colour indicates % relative metabolic activity, with lighter colours indicating less activity and, therefore, stronger impact of the treatment (each bar represents the mean with error bars representing ± SD, *n* = 5–6; one‐way ANOVA followed by a Tukey's HSD, different letters denote significant differences between treatments). (B) *P. syringae* bacterial growth assay. Measurements taken every 30 min for a total of 18 h. Insert: endpoint OD_600_ measurements were compared for significant differences between treatments (each bar represents the mean with error bars representing ± SD, *n* = 5; one‐way ANOVA followed by a Tukey's HSD, different letters denote significant differences between treatments).

Since ISR is generally defined as a broad‐spectrum resistance, we tested the anti‐microbial scope of CP and FP. We first tested another fungal pathogen, *Fusarium virguliforme*, a soilborne pathogen responsible for soybean sudden death syndrome (SDS). Similarly to *B. cinerea*, both FP and CP showed dose‐dependent anti‐fungal activity against *F. virguliforme*. In addition, their synergistic effect was also observed when used in combination (Figure 5A).

To evaluate whether these compounds also display antimicrobial activity against a bacterial pathogen, we assessed their effect against *Pseudomonas syringae* pv. *tomato* DC3000 (Figure 5B). Treatment with both FP and CP alone as well as in combinations had less pronounced effects on bacterial growth, and no significant additive or dose dependent effects were observed with the concentrations tested.

### 2.5 Both HCAA Biosynthesis and Transport Are Required for Their Effect Against *B. Cinerea*

**Figure 6:**
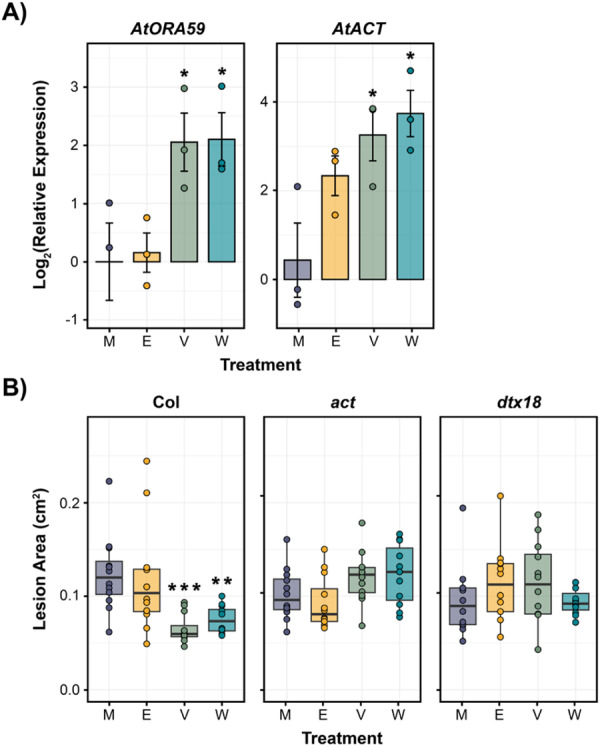
HCAA biosynthesis and transport is required for ISR‐induced resistance against *Botrytis cinerea*. (A) Log_2_ transformed relative expression of *AtORA59* and *AtACT* transcript levels in bacteria‐treated plants compared to MgSO_4_. Transcript levels of tested genes were normalised relative to the reference genes *AtEF1α* and *AtGAPC2* (each bar represents the mean ± SE, *n* = 3; ***p < 0.001, **p < 0.01, *p < 0.05. ANOVA followed by post hoc Dunnett's test was used to assess significant against MgSO_4_ treated samples). (B) Boxplot of *B. cinerea* lesion development on bacterial‐pretreated *Arabidopsis thaliana* mutants (*n* = 12–16; 0 *** < 0.001 ** < 0.01 * < 0.05. ANOVA followed by post hoc Dunnett's test was used to assess significant against MgSO_4_ treated samples). The boxes represent the 25th to 75th percentile, with the 50th percentile indicated by the black line (median). Whiskers extend to 1.5 times the interquartile range (IQR). Points are an overlay of individual data values for the replicates. M—MgSO_4_, E—*E. coli*, V—VFb49, W—WCS417r. [Color figure can be viewed at wileyonlinelibrary.com]

To further understand the metabolic rewiring surrounding the increase in FP and CP synthesis, we explored the transcriptional changes regulating the process. Given the limited genetic data and only preliminary in vitro evidence for the enzymes involved in FP and CP biosynthesis in tomato (Roumani et al. 2025), we utilised Arabidopsis for further investigation. The Arabidopsis agmatine coumaroyl transferase AtACT (At5g61160) catalyzes the final step in the biosynthesis of many HCAAs, including p‐coumaroyl agmatine, FP, and CP, with its overexpression shown to increase pathogen and herbivores resistance (Muroi et al. 2009; Muroi et al. 2012; Dobritzsch et al. 2016). *AtACT* expression is regulated by the JA‐responsive transcription factor AtORA59 (Li et al. 2018a), which is upregulated under ISR primed conditions (Pangesti et al. 2016). Thus, to see whether *AtACT* and *AtORA59* are transcriptionally induced during ISR‐priming, we treated Arabidopsis plants with either *Pseudomonas simiae* WCS417r (Van Wees et al. 1997), as WCS374r is a less effective inducer of ISR in Arabidopsis (Van Wees et al. 1997), and VFb49 [that we had shown to induce ISR in both tomato and Arabidopsis (Loranger et al. 2025)] along with *E. coli* or MgSO_4_. One‐week postpriming, leaf tissue was collected, and reverse transcription quantitative PCR (RT‐qPCR) was conducted. Both *AtORA59* and *AtACT* transcript levels were significantly upregulated in plants treated with WCS417r and VFb49 compared to the MgSO_4_ treatment, while the *E. coli* treatment did not induce significant changes (Figure 6A), suggesting that the upregulation of *AtACT* likely follows the establishment of ISR and may play a pivotal role in ISR related HCAA accumulation. Further, its transcriptional regulation during ISR is likely controlled through the transcription factor AtORA59. This data suggests that the observed ISR‐related HCAA accumulation is not specific for tomato but rather a conserved mechanism in plants.

Thus, to evaluate the importance of HCAA accumulation in ISR‐mediated defence, we tested two Arabidopsis mutants, *act* (SALK_097380) and *dtx18* (SALK_062231). The *act* line is deficient in HCAA synthesis, while *AtDTX18* encodes a multidrug and toxin extrusion (MATE) transporter, which typically facilitates the transport of HCAAs from the cytosol to the apoplast, where it can then act as an anti‐fungal agent (Dobritzsch et al. 2016). Plants were soil‐drenched and incubated for 1 week before being challenged with *B. cinerea*. Disease lesions were measured 4‐day postinfection. As expected, *B. cinerea* lesions were reduced in WCS417 and VFb49‐treated Col‐0 wild type plants, while in both mutant lines the lesion diameter was comparable to the MgSO_4_ control, indicating that not only the production of CP and FP (and possibly other HCAAs) is a key factor in ISR, but their apoplastic localisation plays an equally important role.

## 3 Discussion

*Bacillus* and *Pseudomonas* are two of the best‐studied bacterial genera in the context of plant‐beneficial microbe interactions. Many strains belonging to these genera display either PGPR (Beneduzi et al. 2012; Kumar et al. 2011; Praveen Kumar et al. 2012; Dorjey et al. 2017; Kashyap et al. 2019) or ISR‐inducing capabilities (De Vleesschauwer et al. 2008; Akram et al. 2013; Pieterse et al. 2020; Suresh et al. 2022; Dutilloy et al. 2024). We recently identified a taxonomically diverse set of bacterial strains that induce enhanced resistance in tomato against *B. cinerea* (Loranger et al. 2025). Using one of the strains from that study, the Gram‐positive *B. velezensis* VFb49, and the well‐characterised Gram‐negative *P. defensor* WCS374r, we demonstrated that these strains preferentially adhere to different regions of the root, suggesting strain or species‐dependent interactions with the root surface (Xue et al. 2025). Thus, for the present study, we also used these two strains to identify both strain‐specific and common metabolic changes upon bacterial colonisation of the root. Since multiple studies already looked at metabolomic changes in primed plants after a subsequent pathogen infection (Carlson et al. 2019; Mashabela et al. 2023b; Hossain et al. 2024), our goal here was to look at metabolome changes after ISR induction but prior to a subsequent pathogen infection, i.e. metabolites that are increased directly by ISR priming.

Overall, the numbers of DAMs were relatively small, which was not surprising since the previous transcriptome analyses had shown fairly small changes in non‐challenged plants after ISR induction (Verhagen et al. 2004; Nguyen et al. 2020). We observed 40 features with differential accumulation for tomatoes primed with VFb49 and 29 for those primed with WCS374r. Interestingly, the number of metabolites which increased in abundance was smaller than those that decreased, while for our control, *E. coli*, 9 out of 11 DAMs showed an increase. Presumably, the DAMs in the *E. coli* samples reflect a general stress response or pattern‐triggered immunity (PTI), which might be supressed by beneficial bacteria (Stringlis et al. 2018; Tzipilevich et al. 2021). Only few of the DAMs from the primed plants overlapped with the *E. coli* data set, while 16 DAMs overlapped between the two ISR‐inducing strains. We observed a larger number of DAMs for VFb49 compared to WCS374r. This difference likely reflects either the different strategies of colonisation of these bacteria (Xue et al. 2025) or differences in the recognition by the host plant, as these bacteria produce different extracellular metabolites. Pseudomonads like WCS374r may more actively suppress host detection or evade recognition, leading to a weaker metabolic response while Bacilli trigger a stronger immune response (Stringlis et al. 2018; Teixeira et al. 2021; Tzipilevich et al. 2021). VFb49 may also trigger a broader response due to lifestyle differences, like becoming endophytic (Loranger et al. 2025). The shared DAMs between VFb49 and WCS374r likely represent shared metabolic changes of the above‐ground plant in response to ISR priming.

Two of the three compounds that increased in abundance in primed plants were HCAAs (CP and FP). HCAAs have been implicated in a wide range of processes and responses, ranging from flowering to immunity (Li et al. 2018; Liu et al. 2022). These compounds accumulate after pathogen infection (Zacarés et al. 2007; Muroi et al. 2009; López‐Gresa et al. 2016), and frequently increase in resistant plant cultivars (Newman et al.^2001^; Mashabela et al. 2023a), suggesting their direct role in improving pathogen resistance. They have been suggested to contribute to plant immunity either directly, through antimicrobial capacity, or indirectly, through the strengthening of the cell wall (Yogendra et al. 2015; Zeiss et al. 2021). Different plant species produce different HCAAs and many different types of conjugates have been documented (Li et al. 2018b; Roumani et al. 2021). While p‐coumaroyl agmatine is the predominant HCAA in Arabidopsis (Muroi et al. 2009), CP was the major HCAA in our analysis, which is in line with previous reports in tomato (Roumani et al. 2021, 2022; 2025). Intriguingly, we identified both trans and cis isomers of CP and FP (Supporting Information S1: Figure S2). While the trans isomers are the predominant forms in nature, cis isomers for many HCAAs have also previously been identified (King and Calhoun 2005; Li et al. 2018b). Cis isomers are thought to arise via photoisomerization upon exposure to ultraviolet (UV) radiation. Although trans isomers are recognised as the bioactive configuration, cis isomers may also possess antimicrobial properties (Lambruschini et al. 2020).

Both FP and CP exhibited significant anti‐microbial activity at concentrations of 1–2 mM in this study. This effect was markedly enhanced when FP and CP were combined, resulting in inhibition of spore germination, germ tube growth and mycelium growth of *B. cinerea*. Direct antimicrobial activity of some HCAAs has been reported previously, primarily for agmatine and tyramine conjugates. For example, Muroi et al. (2012) showed increased resistance against *B. cinerea* in *Torenia hybrida* plants overexpressing *AtACT* as well as direct antimicrobial activity of the major HCAA produced by AtACT, p‐coumaroyl agmatine. Similarly, the Arabidopsis *act* mutant displayed increased susceptibility to *Alternaria brassicicola* (Muroi et al. 2009). Inhibition of *Phytophthora infestans* spore germination, but not mycelial growth, by p‐coumaroyl agmatine was also reported (Dobritzsch et al. 2016). The effective concentration of CP and FP confirmed in this study (1–2 mM) aligns well with these previous findings against the oomycete, *P. infestans*, and the fungal pathogens, *A. brassicicola, B. cinerea and Colletotrichum camelliae* (Muroi et al. 2009, 2012; Dobritzsch et al. 2016; Wang et al. 2023). Previous studies also reported endogenous concentrations of these metabolites in different plant species. In Arabidopsis and potato, p‐coumaroyl agmatine can accumulate up to 30 nmol g^−1^ fresh weight (FW) and FP around 0.25 nmol g^−1^ FW (Muroi et al. 2009; Dobritzsch et al. 2016). López‐Gresa et al. 2016 reported increases in CP and FP in virus‐infected tomato reaching up to 1.5 and 7 mmol g^−1^ FW, respectively. Similarly, Chen et al. (2006) showed CP accumulation of up to 5 mg g^−1^ dry weight (DW). Given the strong defence response against pathogenic organisms, these reported endogenous concentrations are likely much higher than those found in plants primed by beneficial bacteria. Indeed, in our study, we observed a twofold increase of CP and FP in primed plants, whereas pathogen infection has been associated with 15–20‐fold increases (Muroi et al. 2009; López‐Gresa et al. 2016). This observation aligns well to the concept of ISR priming, which involves elevating the baseline defence level prior to pathogen attack; ISR‐primed plants can, therefore, mount a faster response upon pathogen detection (Pieterse et al. 2014).

Two of the tomato *SlACT* genes that encode the enzymes which catalyze the final step of HCAA biosynthesis are pathogen‐inducible (Roumani et al. 2025), and while an increase in HCAAs in rhizobacteria‐treated rice roots has been reported (Valette et al. 2020), so far, there is no indication that HCAAs are transported from root to shoot (Roumani et al. 2020; Liu et al. 2022). In addition, considering the inability of the *act* mutant plants to mount an ISR against *B. cinerea* in this study, it is unlikely that the observed HCAAs would be derived from the bacteria; however, we cannot completely rule out that possibility. Thus, the most plausible interpretation is that after ISR priming HCAA metabolism is activated or altered in above‐ground tissues and confers pathogen resistance systemically.

To exert their antimicrobial activity, in Arabidopsis HCAAs are transported into the apoplast by MATE transporters, specifically AtDTX18, as the Arabidopsis *dtx18* knockout lines failed to accumulate high levels of p‐coumaroyl agmatine, FP and CP in the apoplast (Dobritzsch et al. 2016). Furthermore, transgenic potato plants overexpressing *AtACT* and *AtDTX18* together led to an increase in their apoplastic concentration and enhanced resistance against *Phytophthora infestans* (Dobritzsch et al. 2016). The importance of the secretion of the produced HCAAs into the apoplast is strongly supported by the failure of primed *dtx18* mutant plants to mount ISR in this study.

In Arabidopsis, the regulation of many HCAAs depend on the phytohormones JA and ET, acting through the transcription factor AtORA59 (Li et al. 2018a). AtORA59 not only governs the biosynthesis of these compounds by regulating *AtACT* expression but also controls their transport to the apoplast via the regulation of the MATE transporter *AtDTX18* (Li et al. 2018a). Furthermore, *AtORA59* is upregulated in ISR‐primed plants (Pangesti et al. 2016). Our transcriptional analysis confirmed that ISR‐priming by two beneficial strains upregulated *AtORA59* and *AtACT*, supporting the notion that HCAA biosynthesis is transcriptionally activated as part of the ISR response. The importance of this pathway is underscored by the fact that both *act* and *dtx18* mutants were unable to mount ISR against *B. cinerea*, suggesting that compounds such as CP and FP accumulate and subsequently are transported to the apoplast to provide anti‐fungal protection. These finding suggest that ISR is not only defined by a stronger transcriptional response upon subsequent pathogen infection but also relies on pre‐challenge metabolic rewiring that results in an increased accumulation of HCAA‐based antifungal compounds.

**Figure 7:**
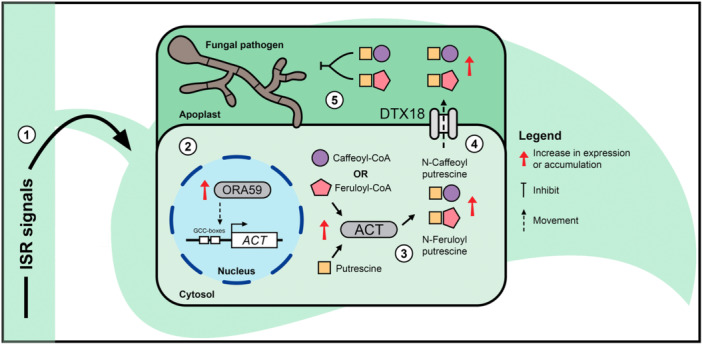
Proposed model of ISR‐induced HCAA accumulation. ISR signals initiated by root‐associated bacteria (1) enhance expression of the transcription factor *ORA59* in the above ground tissues (2). ORA59 directly upregulates *ACT*, promoting cytosolic biosynthesis of N‐Feruloyl putrescine and N‐Caffeoyl putrescine (3), which are then both transported to the apoplast by DTX18 (4). This pre‐infection accumulation of apoplastic HCAAs provides enhanced immunity by acting synergistically to directly restrict the spread of any potential fungal pathogens (5).

While the accumulation of HCAAs have been previously demonstrated in rice roots following beneficial interactions with PGPRs (Valette et al. 2020), or after subsequent infection (Mashabela et al. 2023a), we show here that HCAAs accumulate in above‐ground tissues following priming with ISR‐inducing rhizobacteria prior to a pathogen attack. Further, we demonstrate that in Arabidopsis this accumulation may be linked to transcriptional changes mediated through JA and ET pathways via AtORA59, and that these compounds exhibit strong antifungal activity. Figure 7 summarises the result of this study and the proposed model. Overall, our study demonstrates that different ISR‐inducing bacterial strains have both unique and shared effects on the plant metabolome. Collectively, this work provides novel insights into plant‐microbe interactions and highlights how ISR provides plant protection through the accumulation of HCAAs prior to subsequent infection.

## 4 Methods

### 4.1 Plant Growth Conditions

*Solanum lycopersicum* cv. ‘Glamour’ (tomato) seeds were surface sterilised and then germinated on ½ Murashige and Skoog (MS) 1% sucrose media. Five‐day‐old seedlings were then moved to peat pellets (Jiffy Group, Zwijndrecht, the Netherlands.) and inoculated with target strains, as described below. The tomato plants were grown in a growth chamber with 16 h light cycle, 22°C during the day and 20°C during the evening. *Arabidopsis thaliana* Col‐0 plants were grown in a growth chamber at 22°C with an 8‐h light cycle. The *act* (SALK_097380) and *dtx18* (SALK_062231) lines were obtained from the Arabidopsis Biological resource centre (https://abrc.osu.edu/).

### 4.2 Microbial Culturing and Plant Priming

Bacteria were cultured overnight in Lysogeny Broth (LB) at 28°C. The bacteria were spun down, washed and resuspended in 10 mM MgSO_4_ to an OD_600_ of 0.1. For tomato, 50 mL of the bacterial suspension was added to each peat pellet, once the inoculum was absorbed the seedlings were transferred to the pellets. Three‐week‐old Arabidopsis was soil drenched with 50 mL of bacterial inoculum at an OD_600_ of 0.1. The above ground tissue of the young tomato plants was collected 2‐week postinoculation.

### 4.3 Untargeted Metabolomics

Untargeted metabolomics was performed by Platform Genetics (Vineland Research & Innovation Centre). Unless otherwise noted, all chemicals and standards were purchased from Sigma (> 95% purity). Ultra performance liquid chromatography‐mass spectrometry (UPLC‐MS) grade methanol, acetonitrile, water, 2‐isopropanol, and formic acid were purchased from Fisher Scientific.

Tomato tissue was lyophilised and ground by pestle under liquid nitrogen. Lyophilised, ground (powdered) tomato leaf tissue was extracted with 2 mL chilled acidified 75:25 methanol/water (w/0.1% formic acid, 0.001 mg/mL tryptophan‐d5 as an internal standard) on ice. Samples were vortexed, sonicated for 5 min (twice with vortex between), and centrifuged at 14 000*g* for 1 min. The supernatant was filtered through 0.2‐micron WWPTFE MS membrane filters (Acrodisc) and transferred to LC‐certified vials (Waters, Manchester, UK) or diluted in 250 µL glass inserts (Chromatographic Specialties Inc.). Quality control pools consisting of equal amounts of individual extracted samples were prepared in LC‐certified vials (Waters).

Chromatographic separation was performed on an Acquity I‐Class UPLC (Waters) equipped with an Acquity UPLC BEH C18 1.7 μm column (Waters) using a binary solvent mixture consisting of solvent A (5% acetonitrile in water with 0.1% formic acid) and solvent B (acetonitrile with 0.1% formic acid), with 0.5 μL sample injection at an infusion flow rate of 0.3 mL/min. The gradient was set for solvent B at 0%–40% for 0.0–22.5 min, 100% over 23.0–24.5 min, decreasing to 0% over 25.0–26.0 min (Rogachev and Aharoni 2012). Analytic data were acquired on a Xevo G2‐XS QTOF (Waters) with a capillary voltage of 3 kV (ESI+) and sample cone voltage of 40 eV, with desolvation temperature of 500°C, cone gas flow of 50 L/h, and desolvation gas flow of 800 L/h. MS^E^ data were acquired in positive electrospray ionisation (ESI) mode. A survey scan time of 0.25 s in continuum data format with an acquisition mass range of 50–1200 Da was used for MS^E^. MS^E^ low collision energy was 6 eV and high ramp collision energy 15–35 eV. Raw MS data and theoretical m/z were assessed and calculated in MassLynx 4.1 (Waters). Leucine enkephelin (200 pg/μL in 50:50 acetonitrile/water with 0.1% formic acid) was used as a reference calibrant with LockSpray ion source (Waters; infusion flow rate 10 μL/min) for exact mass measurement. Mass errors were calculated using the formula: (observed m/z − theoretical m/z)/(theoretical m/z × 100).

Peak alignment, quality control assessment, peak picking, pseudomolecular ion (adduct) composition (including sodium and water adducts), and normalisation to all mass features was performed in Progenesis QI (Non‐Linear Dynamics). Mass features in untargeted data were processed with background/low abundance peaks filtered out (coefficient of variation < 50% in pooled QCs and manual processing of background ions and molecular ions of interest in raw data), then annotated for molecular possibilities within 5 ppm (> 200 m/z) or 2 mDa (< 200 m/z) using an elemental range of C0‐200, H0‐400, N0‐20, O‐60, S0‐3, Na0‐1 in a customised in‐house natural products (microbes and plant) database founded on the PlantCyc (Hawkins et al. 2025) database and COCONUT (Chandrasekhar et al. 2025).

The mass features above were compiled after noise/background filtering of Progenesis QI (PQI)‐processed raw data (normalised to all ions detected) with neutral mass predictions composed considering commonly observed [M + H], [M + Na], [M + H‐H2O] adducts for ESI+ . Raw data for all timepoints were imported together for processing in PQI. Quality control passed using pooled QCs, internal standard, and PCA. Data annotation and identification from LC‐MS based metabolomics was based on PlantCyc (Hawkins et al. 2025) and the KNApSAcK comprehensive species‐metabolite relationship database (Shinbo et al. 2006).

### 4.4 *Botrytis Cinerea* Challenge Assay

*Botrytis cinerea* isolate MEE B191, was used for this study (provided by Canadian Collection of Fungal Cultures, Agriculture and Agri‐Food Canada, Ottawa, ON, Canada). *B. cinerea* was grown on potato dextrose agar (PDA) at 21°C in darkness for 7 days. The inoculum was prepared by collecting fungal conidia in Sabouraud maltose broth (SMB), which was then adjusted to a conidial density of 2.5 × 10^5^/mL for tomato and 2.5 × 10^6^/mL for Arabidopsis. For treatments that included compounds, they were initially suspended in SMB to a concentration of 10 mM and then diluted into the fungal suspension to the final desired concentration. Four‐week‐old tomato plants were inoculated by placing a 10 μL droplet of fungal suspension on the adaxial midvein of detached leaflets. The inoculated leaves were kept sealed in Petri dishes lined with water‐soaked sterile filter paper to maintain high humidity. Arabidopsis leaves were inoculated directly, without being detached, with a 10 μL droplet of fungal suspension applied to adaxial midvein of the selected leaves. The containers with the plants were then domed and sealed to maintain high humidity. Petri dishes and domed Arabidopsis plants were kept at room temperature and under 24 h light conditions for 3–4 days. Images were taken and lesion area was analyzed using the Colour‐analyzer tool (Loranger et al. 2024).

### 4.5 *Pseudomonas Syringae* Growth Assay

*Pseudomonas syringae* pv. *tomato* DC3000 was cultured overnight at 28°C in King's B (KB) media. The following day the culture was spun down, washed with 10 mM MgCl_2_ and adjusted to an OD_600_ of 0.1. 150 μL of this suspension was added to wells of a 96‐well plate. Compounds were suspended in ddH_2_O to a concentration of 10 mM and diluted into the wells to reach the final desired concentrations. A no‐treatment control was supplemented with ddH_2_O for growth comparison. The plate was sealed and grown in a TECAN Infinite M1000 Pro at 28°C with OD_600_ reading taken every 30 min for a total of 18 h. Final OD measurements were blanked to a no‐bacterial control.

### 4.6 In Vitro Fungal Growth Inhibition

*B. cinerea* and *Fusarium virguliforme* were added separately to 96‐well plates at a conidial density of 2.5 × 10^4^/mL, in a final volume of 150 μL. SMB media with a pH of 6.6 was used for this in vitro work as the traditional SMB pH of 5.6 is considered to acidic, as resazurin is pH sensitive. Compounds were suspended in SMB pH 6.6 to a concentration of 10 mM and were diluted into the wells to reach the final desired concentrations. 96‐well plates were incubated at room temperature for 24 h, at this point 15 μL SMB pH 6.6 supplemented with 3 mg/mL of resazurin was added to each well. Plates were read 16 h later (40 h final time point) using a TECAN Infinite M1000 Pro (excitation 530 nm, emission 590 nm). Compound treatments were normalised to a no‐compound control set as 0% inhibition of fungal growth and a no‐fungus control as 100% inhibition.

Imaging of conidial germination was done using a Leica DFC7000 T microscope camera. Conidia were suspended to a density of 2.5 × 10^5^/mL in SMB media. Compounds were suspended in SMB media and diluted appropriately in the conidial suspension to reach the desired concentrations. Conidia were incubated for 24 h before being imaged. Conidial germination and germ tube length were measured based on images taken using Fiji (Schindelin et al. 2012).

### 4.7 Gene Expression Analysis

Leaf tissue was harvested from 4‐week‐old Arabidopsis plants. Total RNA was extracted using the RNeasy Plant Mini Kit (QIAGEN), following the manufactures instructions. The cDNA was synthesised using the iScript cDNA synthesis kit (Bio‐Rad). qPCR was conducted using the PowerTrack SYBR Green Master Mix (Applied Biosystems) and the CFX96 Touch Real‐Time PCR Detection System (Bio‐Rad). Primer sequences are provided in Supporting Information S2: Table S6. The primers used for the quantification of *AtACT* (AT5G61160) were first described in Muroi et al. 2012 and the primers for *AtORA59* (AT1G06160) first described in Pangesti et al. 2016. *AtGAPC2* (AT1G13440) and *AtEf1α* (AT5G60390) were used as reference genes. The relative expression of each gene compared to the MgSO_4_ samples was calculated using a modified Pfaffl equation (Pfaffl 2001) to incorporate both primer efficiency and multiple reference genes. The resulting relative expression was then log_2_ transformed. Each treatment group was made up of three biological replicates, each containing three technical replicates.

### 4.8 Data Analysis

The untargeted metabolomics data was analyzed using a two‐stage statistical approach. First a one‐way ANOVA was conducted for each of the metabolic features to identify the ones that showed significant variation across treatment groups. The *p*‐values were adjusted for multiple testing using the FDR method (Benjamini & Hochberg, BH). A total of 94 of the 4065 were found to be significant (*q*‐value < 0.05) after the first stage of filtering. A post hoc Dunnett's test was then used to compare each treatment against the MgSO_4_ control while again controlling for the multiple comparisons through an FDR adjustment. Features were then filtered for *q*‐value < 0.05 and an absolute log_2_FC > 0.5849 (1.5‐fold). Statistical tests and plotting were performed using RStudio. PCA loading values were extracted using the RStudio package FactoMineR (Lê et al. 2008). Unless otherwise specified, significance was calculated using a one‐way ANOVA followed by a Dunnett's post hoc test comparing against the mock or no‐treatment control.

### 4.9 Accession Numbers

*AtACT* (At5g61160), *AtORA59* (AT1G06160), *AtDTX18* (AT3G23550).

## Ethics Statement

The authors have nothing to report.

## Conflicts of Interest

The authors declare no conflicts of interest.

## Supporting information


**Supplemental Figure 1:** MS fragmentation for the feature putatively identified as N‐Caffeoyl putrescine.
**Supplemental Figure 2:** MS Chromatogram and quantification of FP and CP isomers.

**Supplemental Table 1:** Summary of VFb49‐induced DAMs with loading values.
**Supplemental Table 2:** Summary of WCS374r ‐induced DAMs with loading values.
**Supplemental Table 3:** Summary of E. coli‐induced DAMs with loading values.
**Supplemental Table 4:** Overlapping bacterial responsive DAMs.
**Supplemental Table 5:** Compounds related to Caffeoyl/Feruloyl putrescine biosynthesis.
**Supplemental Table 6:** RT‐qPCR primers.

## Data Availability

The datasets generated and analyzed during the current study are available from the corresponding author on request.
