# Supplementary material for: Rhizobacteria‐Induced Systemic Priming Against Fungal Pathogens Involves Hydroxycinnamic Acid Amides
Source: Plant Cell Environ. 2026 Mar 30;49(8):4907–21. doi: 10.1111/pce.70495 (PMC13353667; doi:10.1111/pce.70495)
Supplement: Supplementary file 1 — Supplemental Figure 1: MS fragmentation for the feature putatively identified as N‐Caffeoyl putrescine. Supplemental Figure 2: MS Chromatogram and quantification of FP and CP isomers. [file PCE-49-4907-s001.pptx]

## Slide 1
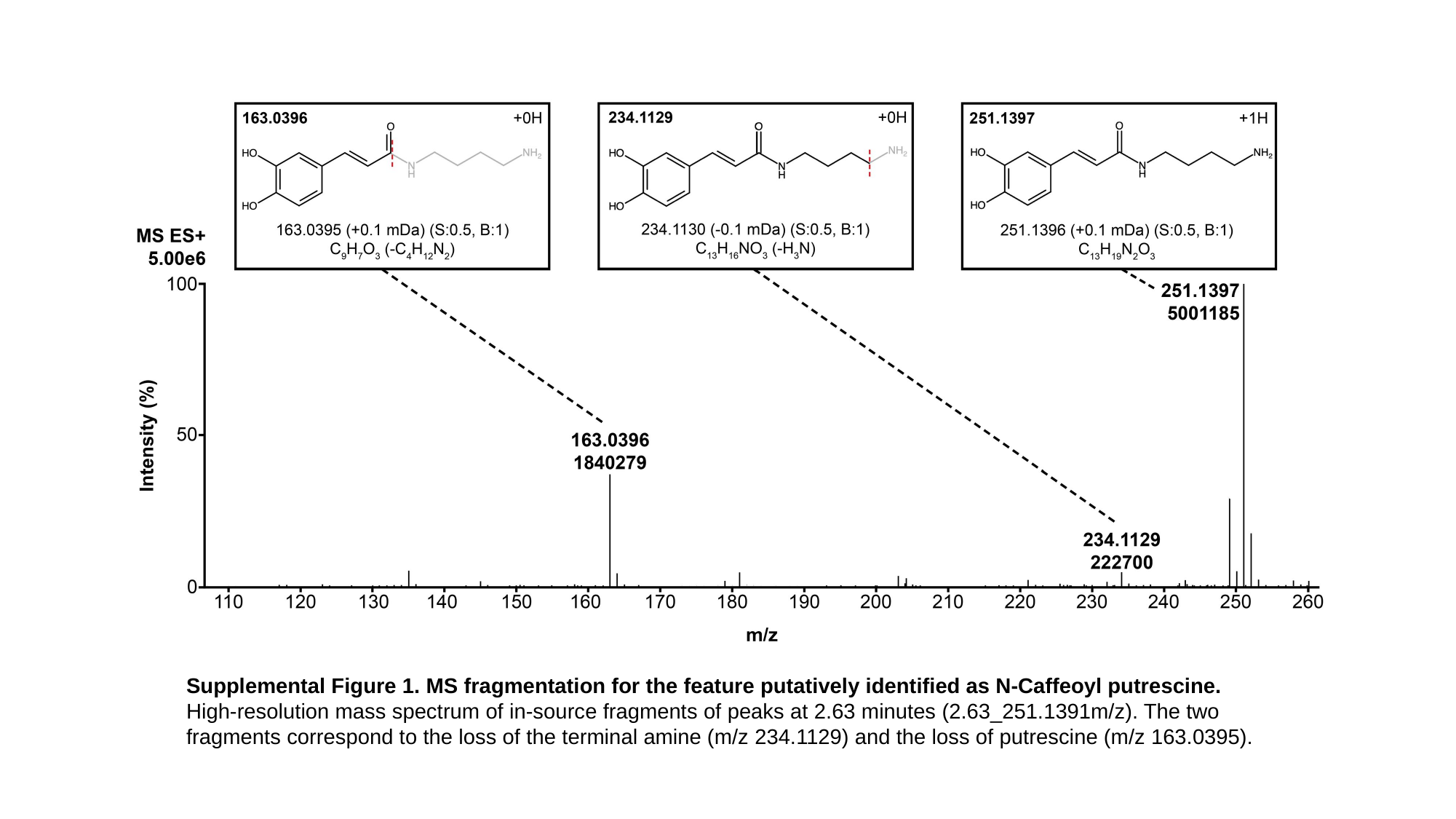

Supplemental Figure 1. MS fragmentation for the feature putatively identified as N-Caffeoyl putrescine.
High-resolution mass spectrum of in-source fragments of peaks at 2.63 minutes (2.63_251.1391m/z). The two fragments correspond to the loss of the terminal amine (m/z 234.1129) and the loss of putrescine (m/z 163.0395).

## Slide 2
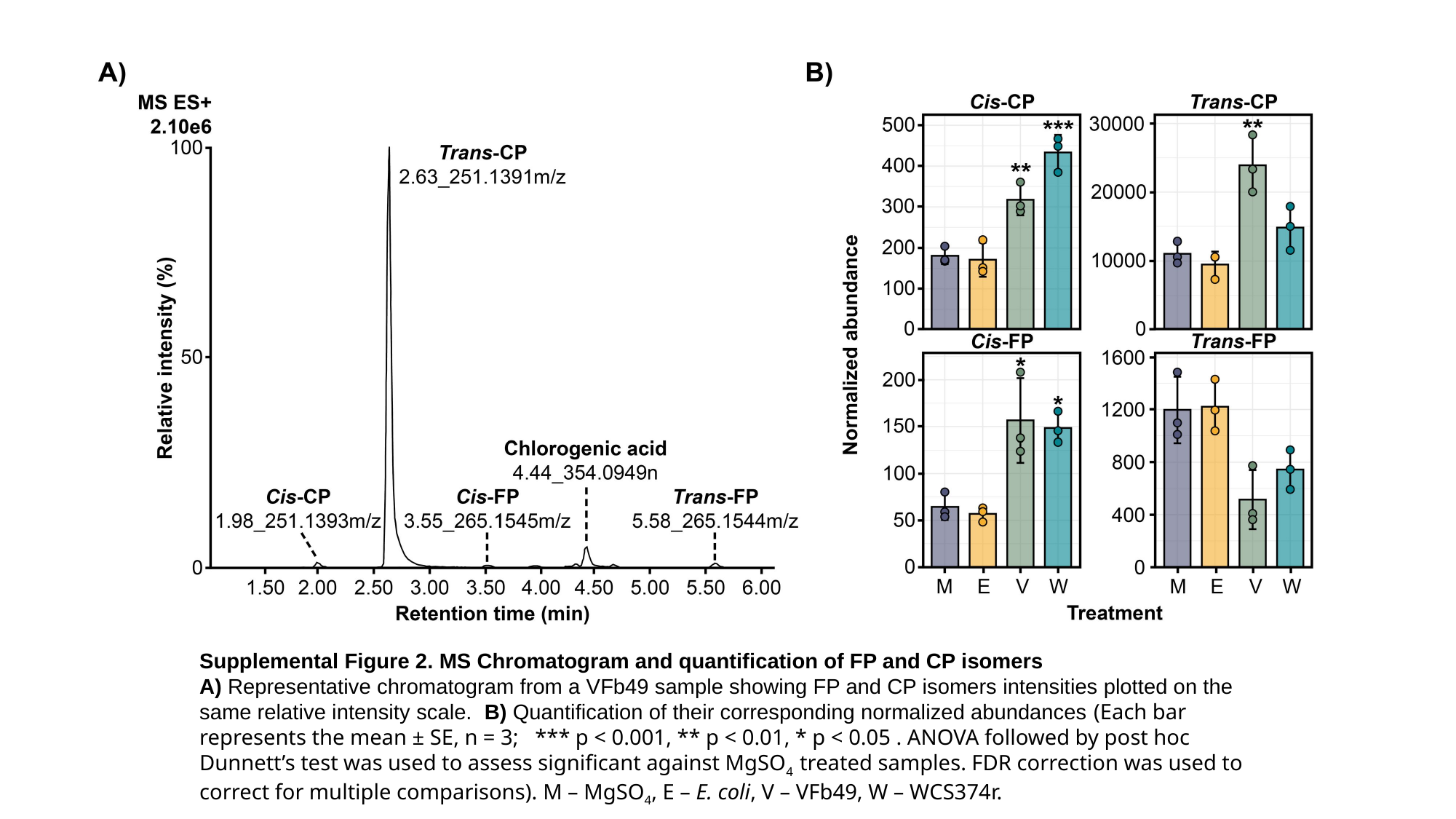

Supplemental Figure 2. MS Chromatogram and quantification of FP and CP isomers
A) Representative chromatogram from a VFb49 sample showing FP and CP isomers intensities plotted on the same relative intensity scale. B) Quantification of their corresponding normalized abundances (Each bar represents the mean ± SE, n = 3; *** p < 0.001, ** p < 0.01, * p < 0.05 . ANOVA followed by post hoc Dunnett’s test was used to assess significant against MgSO4 treated samples. FDR correction was used to correct for multiple comparisons). M – MgSO4, E – E. coli, V – VFb49, W – WCS374r.
