# Supplementary material for: Rhizobacteria‐Induced Systemic Priming Against Fungal Pathogens Involves Hydroxycinnamic Acid Amides
Source: Plant Cell Environ. 2026 Mar 30;49(8):4907–21. doi: 10.1111/pce.70495 (PMC13353667; doi:10.1111/pce.70495)
Supplement: Supplementary file 2 — Supplemental Table 1: Summary of VFb49‐induced DAMs with loading values. Supplemental Table 2: Summary of WCS374r ‐induced DAMs with loading values. Supplemental Table 3: Summary of E. coli‐induced DAMs with loading values. Supplemental Table 4: Overlapping bacterial responsive DAMs. Supplemental Table 5: Compounds related to Caffeoyl/Feruloyl putrescine biosynthesis. Supplemental Table 6: RT‐qPCR primers. [file PCE-49-4907-s002.pdf]

Supplemental Table 1. Summary of VFb49-induced DAMs with loading values

Metabolite	Log2FC	q-value	Putative ID	abs(PC3 LV)
14.37_596.2375m/z	0.68349961	0.01999313	-	0.82426588
4.51_304.1390m/z	-0.6495354	0.032179	Tyrosine betaine	0.7397513
19.43_1090.5340m/z	-1.0996564	0.00648434	-	0.666568845
4.56_411.1257m/z	4.59554599	0.00113559		0.64990883
7.89_365.0839m/z	4.88417259	0.00147094	1-O-Caffeoyl-beta-D-glucose	0.63233476
4.91_234.5634m/z	5.22185125	0.02553341	-	0.62987898
16.45_422.7371m/z	-0.743196	0.03336466	-	0.621910652
2.19_521.9683m/z	-0.6214827	0.03536327	-	0.6187156
3.48_235.1461m/z	0.88586361	0.00176797	-	0.584946978
10.69_431.0872m/z	3.69578409	0.00385952	-	0.58275392
10.71_354.1745m/z	2.06197281	0.00228777	-	0.57531949
10.69_359.1305m/z	2.29752654	0.00346769	Steroid O sulfate	0.56700489
20.27_819.3067m/z	1.1687478	0.01126385	-	0.552530119
2.63_251.1391m/z	1.11252536	0.00452367	N-Caffeoyl putrescine	0.52731777
2.63_234.1120m/z	1.09346347	0.00386935	N-Caffeoyl putrescine (frag.)	0.52062774
21.19_842.4665m/z	-3.6690942	0.00121421	-	0.510147
2.65_266.1266n	1.04736965	0.00541824	-	0.510088028
2.65_204.1016m/z	1.16660349	0.0046355	Indole-3-butyrate	0.50627596
2.65_221.1254m/z	0.76300637	0.0032006	-	0.44557003
2.41_252.0728m/z	0.99250221	0.00434683	-	0.3710059
1.34_86.0603m/z	0.59897674	0.00214527	-	0.368875487
2.06_287.1232m/z	4.43790564	0.00011416	2'-Deoxymugineate	0.34026551
1.69_513.9978m/z	-1.0311066	0.01128887	-	0.2976464
1.90_259.1288m/z	1.21020957	0.00490913	L-Saccharopine	0.2755077
21.34_901.4774m/z	-0.8649581	0.04162341	-	0.21271256
7.15_211.1435m/z	4.30995443	0.04635673	7,8-Diaminopelargonate	0.20957618
1.98_251.1393m/z	0.80682277	0.00769209	N-Caffeoyl putrescine	0.196994992
2.19_515.9908m/z	-2.3021903	0.00407745	-	0.15052
8.71_661.3351m/z	-1.9904938	0.00147092	-	0.1460056
3.55_265.1545m/z	1.26977934	0.01372166	N-Feruloyl putrescine	0.13407636
2.58_515.9864m/z	-1.9782502	0.0012007	-	0.1084316
11.09_487.1182m/z	-1.6082713	0.04406462	-	0.072388007
12.45_948.2873n	-1.418395	0.02312572	-	0.0634559
11.09_934.2712n	-1.5314733	0.0343241	-	0.063399813
1.88_313.0891m/z	-1.6779161	0.00306427	-	0.05533541
9.79_951.2743m/z	-0.7669729	0.02509875	-	0.0487526

11.91_965.2881m/z	-1.0059244	0.01007315	-	0.0212907
9.79_789.2216m/z	-0.9663947	0.02963509	-	0.0162197
11.09_479.1194m/z	-1.5640477	0.03564874	Isorhamnetin-3-O-glucoside	0.002122
13.35_979.3061m/z	-0.9829107	0.00601676	-	0.00165621

Supplemental Table 2. Summary of WCS374r -induced DAMs with loading values

Metabolite	Log2FC	q-value	Putative ID	abs(PC3 LV)
14.41_457.2085m/z	0.94381263	0.01929535	-	0.901132822
14.37_596.2375m/z	-1.32305	0.01999313	-	0.82426588
17.59_387.1620m/z	-1.0810898	0.00487279	-	0.65959047
8.75_177.0544m/z	0.75800509	0.00041758	?	0.5205064
17.66_902.5086m/z	-0.726263	0.00037951	-	0.47025139
8.26_181.1221m/z	0.58740076	0.0010313	Dihydroactinidiolide	0.4310318
2.41_252.0728m/z	1.09356691	0.00372441	-	0.3710059
17.66_462.7493m/z	-0.6402082	0.00249701	-	0.36852865
6.11_179.0337m/z	0.93631314	0.00077959		0.3211319
6.11_449.0398m/z	0.94967471	0.00278529	-	0.2873449
1.90_259.1288m/z	1.31077029	0.00470041	L-Saccharopine	0.2755077
21.34_901.4774m/z	-1.6251927	0.0080819	-	0.21271256
1.98_251.1393m/z	1.25468013	0.0001283	N-Caffeoyl putrescine	0.196994992
6.11_452.0033m/z	1.08971741	0.00209858	-	0.1787029
2.19_515.9908m/z	-1.0605285	0.02877063	-	0.15052
8.71_661.3351m/z	-0.7314756	0.03611461	-	0.1460056
3.55_265.1545m/z	1.19269991	0.01372166	N-Feruloyl putrescine	0.13407636
2.58_515.9864m/z	-0.7798075	0.02555401	-	0.1084316
11.09_773.2274m/z	-2.1240936	0.04614127	-	0.0752293
11.09_487.1182m/z	-2.5142098	0.03014592	-	0.072388
12.45_948.2873n	-2.2949386	0.01974542	-	0.063455854
11.09_934.2712n	-2.2413248	0.02711521	-	0.0633998
1.88_313.0891m/z	-2.003146	0.00277178	-	0.05533541
9.79_951.2743m/z	-1.3274692	0.01049544	-	0.0487526
11.09_495.1076m/z	-1.3860794	0.03174489	-	0.0434467
11.91_965.2881m/z	-1.3962444	0.00521492	-	0.0212907
9.79_789.2216m/z	-1.4023386	0.01474653	-	0.0162197
11.09_479.1194m/z	-3.184667	0.01369059	Isorhamnetin-3-O-glucoside	0.002122
13.35_979.3061m/z	-1.3922494	0.00273445	-	0.00165621

Supplemental Table 3. Summary of *E. coli*-induced DAMs with loading values

Metabolite	Log2FC	q-value	Putative ID	abs(PC3 LV)
14.41_457.2085m/z	0.846212455	0.02231013	-	0.901132822
14.37_596.2375m/z	-1.825383308	0.01999313	-	0.82426588
17.59_387.1620m/z	-0.6118645	0.02644842	-	0.659590473
1.25_392.1116m/z	1.150713692	0.00043113	-	0.524038053
21.19_842.4665m/z	0.87461352	0.00141506	-	0.51014704
2.41_252.0728m/z	0.671106805	0.04368814	-	0.371005944
20.32_629.8233m/z	0.634493147	0.00084472	-	0.359112168
2.06_287.1232m/z	4.108466706	0.000697578	2'-Deoxymugineate	0.340265506
2.38_374.2013m/z	0.693975545	0.04570058	-	0.260660773
12.45_948.2873n	0.709203005	0.02312572	-	0.063455854
11.09_495.1076m/z	0.68874105	0.03174489	-	0.043446699

Supplemental Table 4. Overlapping bacterial responsive DAMs

Metabolite	VFb49 Log2FC	WCS374r Log2FC	*E. coli* Log2FC	Putative ID
2.41_252.0728m/z	0.99250221	1.093566914	0.671106805	-

Supplemental Table 5. Compounds related to Caffeoyl/Feruloyl putrescine metabolism in VFb49, WCS374r, *E.coli* datasets

Metabolite	VFb49 Log2FC	q-value	Putative ID
3.55_265.1545m/z	1.26977934	0.01372166[1]	N-Feruloyl putrescine (cis)
1.98_251.1393m/z	0.80682277	0.00769209[1]	N-Caffeoyl putrescine (cis)
5.58_265.1544m/z	-1.217942751	0.061118154[2]	N-Feruloyl putrescine (trans)
2.63_251.1391m/z	1.112595492	0.00452367[1]	N-Caffeoyl putrescine (trans)
5.70_194.0578n	0.738880014	0.077411499[2]	Ferulic acid
6.11_163.0392m/z	0.107302788	0.061285113[2]	Umbelliferone
2.19_180.0423n	-0.447650888	0.061118154[2]	Caffeic acid
7.27_368.1101n	-0.515739064	0.061118154[2]	3-O-Feruloyl-D-quinic acid
2.60_179.0338m/z	0.047238185	0.061118154[2]	Esculetin
1.12_182.0812m/z	-0.000772141	0.254335369[2]	L-Tyrosine

4.44_354.0949n	-0.165229781	0.225060865[2]	Chlorogenic acid
2.09_166.0864m/z	-0.038512299	0.241489745[2]	L-Phenylalanine
5.99_175.0389m/z	0.062995238	0.299682209[2]	Scopoletin
2.58_372.0688n	-0.114466461	0.282618243[2]	2-O-Caffeoylglucarate

[1]q-value from follow up Dunnett's post-hoc test
[2]q-value from initial one-way ANOVA (did not pass significance threshold)

**Metabolite**	**WCS374r Log2FC**	**q value**	**Putative ID**
3.55_265.1545m/z	1.19269991	0.01372166[1]	N-Feruloyl putrescine (cis)
1.98_251.1393m/z	1.25468013	0.0001283[1]	N-Caffeoyl putrescine (cis)
5.58_265.1544m/z	-0.686075465	0.061118154[2]	N-Feruloyl putrescine (trans)
2.63_251.1391m/z	0.423171608	0.48382502[1]	N-Caffeoyl putrescine (trans)
5.70_194.0578n	0.483014732	0.077411499[2]	Ferulic acid
6.11_163.0392m/z	0.63416208	0.061285113[2]	Umbelliferone
2.19_180.0423n	-0.123884871	0.061118154[2]	Caffeic acid
7.27_368.1101n	-0.212037286	0.061118154[2]	3-O-Feruloyl-D-quinic acid
2.60_179.0338m/z	0.335576365	0.061118154[2]	Esculetin
1.12_182.0812m/z	-0.104327523	0.254335369[2]	L-Tyrosine
4.44_354.0949n	-0.151592471	0.225060865[2]	Chlorogenic acid
2.09_166.0864m/z	0.00972081	0.241489745[2]	L-Phenylalanine
5.99_175.0389m/z	0.151685633	0.299682209[2]	Scopoletin
2.58_372.0688n	-0.008542632	0.282618243[2]	2-O-Caffeoylglucarate

[1]q-value from follow up Dunnett's post-hoc test
[2]q-value from initial one-way ANOVA (did not pass significance threshold)

**Metabolite**	***E. coli* Log2FC**	**q Value**	**Putative ID**
3.55_265.1545m/z	-0.1751083	0.96760747[1]	Feruloyl putrescine (cis)
1.98_251.1393m/z	-0.0784898	0.97702655[1]	N-Caffeoyl putrescine (cis)
5.58_265.1544m/z	0.02812253	0.061118154[2]	Feruloyl putrescine (trans)
2.63_251.1391m/z	-0.2195447	0.84895271[1]	N-Caffeoyl putrescine (trans)
5.70_194.0578n	-0.0647328	0.077411499[2]	Ferulic acid
6.11_163.0392m/z	0.03704559	0.061285113[2]	Umbelliferone
2.19_180.0423n	0.16627812	0.061118154[2]	Caffeic acid
7.27_368.1101n	-0.0390312	0.061118154[2]	3-O-Feruloyl-D-quinic acid
2.60_179.0338m/z	-0.0191414	0.061118154[2]	Esculetin

1.12_182.0812m/z	0.28410614	0.254335369[2]	L-Tyrosine
4.44_354.0949n	-0.0208408	0.225060865[2]	Chlorogenic acid
2.09_166.0864m/z	0.23818727	0.241489745[2]	L-Phenylalanine
5.99_175.0389m/z	-0.0474104	0.299682209[2]	Scopoletin
2.58_372.0688n	0.00084786	0.282618243[2]	2-O-Caffeoylglucarate

[1]q-value from follow up Dunnett's post-hoc test
[2]q-value from initial one-way ANOVA (did not pass significance threshold)

Supplemental Table 6. RT-qPCR primers

Gene	FW primer 5' – 3'	RV primer 5' – 3'	Amplicon	Efficiency
AtACT[1]	tcgggacttgtgtcttagcc	cccgtttacgaccaacaact	203bp	92.75%
AtORA59[2]	ttccccggagaactcttctt	gcctgatcataagcgagagc	339bp	101.59%
AtGAPC2	catcccatggggtgaggctgga	atgggcgcatctttgcttgggg	138bp	99.78%
AtEf1α	tgagcacgctcttcttgctttca	ggtggtggcatccatcttgttaca	76bp	97.80%

[1](Muroi *et al.*, 2012) [2](Pangesti *et al.*, 2016)
